# Effect of sodium and calcium on polysaccharide production and the activities of enzymes involved in the polysaccharide synthesis of *Lentinus edodes*

**DOI:** 10.1186/s13568-020-00985-w

**Published:** 2020-03-14

**Authors:** Bilal Adil, Quanju Xiang, Maolan He, Yuetong Wu, Muhammad Ahsan Asghar, Muhammad Arshad, Peng Qin, Yunfu Gu, Xiumei Yu, Ke Zhao, Xiaoping Zhang, Menggen Ma, Qiang Chen, Xiaoqiong Chen, Yanhong Yan

**Affiliations:** 1 College of Resource, Sichuan Agricultural University, Chengdu, 611130 Sichuan People’s Republic of China; 2 Rice Research Institute of Sichuan Agricultural University, Chengdu, 611130 Sichuan People’s Republic of China; 3 Key Laboratory of Crop Ecophysiology and Farming System in Southwest China, College of Agronomy, Sichuan Agricultural University, Chengdu, 611130 Sichuan People’s Republic of China; 4 College of Animal Science and Technology, Sichuan Agricultural University, Chengdu, 611130 Sichuan People’s Republic of China

**Keywords:** *Lentinus edodes*, Sodium, Calcium, Polysaccharides, Enzymatic activity, Gene expression

## Abstract

Lentinan is a *Lentinus edodes* secondary metabolite that can regulate human immune function, but yields are low. Here, the effects of Ca^2+^ and Na^+^ on *L. edodes* lentinan content were investigated. Metal ion concentrations and induction times were optimized according to mycelial biomass, and intracellular polysaccharide (IPS), extracellular polysaccharide (EPS), and total polysaccharide (TPS) content. The activities and gene expression of phospho-glucose isomerase (PGI), phosphoglucomutase (PGM), and UDP-glcpyrophosphorylase (UGP) were also measured. Ca^2+^ and Na^+^ concentration and induction time affected biomass, IPS, and EPS concentrations. Na^+^ increased EPS, IPS and TPS, while Ca^2+^ increased biomass, IPS, and TPS. During fermentation, mycelial biomass varied greatly under Ca^2+^ induction, while IPS, EPS and TPS varied greatly under Na^+^ induction. PGM and UGP activities increased in the presence of Na^+^, while PGI increased with Ca^2+^. Compared to control samples, *pgi* and *pgm* expression under Na^+^ was greater at days 45 and 60, respectively, while under Ca^2+^, *ugp* expression was greater at day 45. IPS content correlated significantly with enzyme activity, while EPS correlated with PGM activity. Our data contributes to better understanding how Na^+^ and Ca^2+^ affect mycelial growth and secondary metabolite production, and of polysaccharide biosynthesis mechanisms of *L. edodes*.

## Introduction

Most commercial mushroom species belong to the *Basidiomycota* and are absorptive, obtaining external nutrients for the growth of vegetative mycelium (Taylor and Ellison 2010). Mushrooms have high nutritional value, including high protein, fiber, and essential amino acids, and low fat content (Carneiro et al. 2013). *Lentinus edodes* is an important edible mushroom that is widely cultivated globally (Jiang et al. 2012). *L.* edodes has high nutritional value and therapeutic properties and can be used in cooking and medicinal applications. Its mycelia and fruiting bodies contain compounds such as carbohydrates, lipids (linoleic acid), protein (26% dry weight), minerals, fibers, vitamins (B1, B2 and C) and ergosterol (Finimundy et al. 2014). Moreover, *L.* edodes is rich in the polysaccharide lentinan, a β-glucan that has been extensively used as anti-metastatic, anti-gingivitis, antifungal, antibacterial, anti-diabetic, antitumor, and high immuno-potentiating agent (Zhang et al. 2011). The by-products of *L.* edodes substrate can also serve as materials for preparation, characterization and antioxidant activity of polysaccharide. However, the low concentration of lentinan in fruiting bodies and mycelia limits its application and popularization.

Growth and secondary metabolite production are affected by many factors, including temperature, pH, and culture conditions (Fan et al. 2007). In one study, cell growth and intracellular polysaccharide (IPS) accumulation of *Phellinus linteus* (*P. linteus*) decreased when NaCl was added to the culture (Zhu et al. 2016). When 2.0 g/L of ascorbic acid was added to the culture broth of *Hericium erinaceus*, melanism was eliminated and mycelial growth was promoted (Lee et al. 2010). Addition of 10 mM Mn^2+^ and Ca^2+^ at the start of a static liquid cultivation of *Ganoderma lucidum* resulted in 2.2- and 3.7-fold increases in total ganoderic acid production, respectively (Xu and Zhong 2012; Xu et al. 2014). Metal ions including Fe^2+^, Zn^2+^, Ca^2+^, Mg^2+^ and Cu^2+^ could inhibit mycelium growth of *Tricholoma mongolicum*, but promoted polysaccharide production (Min et al. 2011). Therefore, production of secondary metabolites can be improved by optimizing conditions, such as by adding metal ions.

Many important enzymes are involved in polysaccharide synthesis. UDPG-pyrophosphorylase (UGP) can convert glucose-1-phosphate to UDP-glucose, while glucose-1-phosphate can be produced by catalyzing the reversible isomerization between glucose-1-phosphate and glucose-6-phosphate via phosphoglucomutase (PGM) (Shingel 2004). At the branching point of the Embden–Meyerhof–Parnas (EMP) pathway, phosphoglucose isomerase (PGI) leads to pyruvic acid formation and PGM leads to polysaccharide formation (Tang and Zhong 2002). These three enzymes play key roles in pathways of metabolite synthesis. Higher activities of PGM, UGP, and PGI were desirable for the biosynthetic rate of IPS in *Cordyceps militaris* (*C. militaris*), which is a member of Ascomycota (Zhu et al. 2016). The activities of PGM and UGP highly correlated with the amount of polysaccharide produced in *Streptococcus thermophilus* (*S. thermophilus*) and *Aureobasidium pullulans* (*A. pullulans*) (Degeest and De Vuyst 2000; Pan et al. 2013). These results indicate that the activities of these enzymes highly affect polysaccharide yield.

There are no reports showing how metals can promote mycelium growth and polysaccharide yield in *L. edodes.* Therefore, in the present study, Na^+^ and Ca^2+^ were chosen to: (i) Investigate the optimal concentration and induction time for production of biomass, and intracellular, extracellular, and total polysaccharides; (ii) To demonstrate the changes in enzymatic activities of three key enzymes (PGM, PGI, and UGP) and their related gene expression after induction via Na^+^ and Ca^2+^.

## Materials and methods

### Experimental strain and culture conditions

The mushroom strain used in this study was *L. edodes* 808 (ACCC 52357), which was obtained from the Chengdu Academy of Agriculture and Forestry Sciences. *L. edodes* was cultured on sterile potato dextrose agar medium (PDA) for 10 days at 25 °C. When plates were fully covered with mycelia, mycelial plugs (5 mm diameter) were used for inoculation. Three mycelial plugs were inoculated into 250 mL Erlenmeyer flasks containing 50 mL synthetic medium (35 g glucose, 5 g peptone, 2.5 g yeast extract, 1 g KH_2_PO_4_·H_2_O, 0.5 g MgSO_4_·7H_2_O, 0.05 g vitamin B1, and 1 L distilled water, sterilized at 121 °C for 30 min), and kept at 28 °C. Five different concentrations (0, 50, 200, 600, and 1000 mg/L) of Na^+^ (NaCl) and Ca^2+^ (CaCl_2_) were used in this study. For the determination of optimal induction time, metals were added to medium at four different time points: (0); at inoculation (1); after 3 days of static culture (2); after shaking (28 °C, 150 rpm) for 7 days (3); static after shaking culture metal ions added after 10 days. Samples were cultured at 28 °C for 50 days in a static incubator and collected for further biomass and polysaccharide determination. Metals were added at the optimal induction concentration and time, and the dynamic changes in biomass, polysaccharide content, enzyme activities, and transcriptional expression were analyzed every 10 days. Experiments were performed in triplicate.

### Determination of biomass

Biomass was obtained by vacuum filtering fermentation broth through a 100-mesh screen, washing the filtrate three times with distilled water, and drying filtrate at 50 °C until weight was constant.

### Determination of EPS and IPS concentrations

To estimate the IPS concentration, 0.2 g of dried mycelium was ground into a powder in the presence of liquid nitrogen. Fifty times the volume of boiling water was added and refluxed for 1 h, with 1 repeat. Samples were then centrifuged at 3000 rpm/min for 30 min at 4 °C. Four volumes of 95% ethanol was added to the supernatant, and then incubated at 4 °C overnight, followed by centrifugation at 3000 rpm/min for 30 min at 4 °C. Excess ethanol was removed by evaporation and the precipitate was dissolved in 2 mL water. Polysaccharide content was measured using the phenol–sulfuric acid method, with glucose as a standard (Dubois et al. 1951; Tang et al. 2009). The fermentation liquid was centrifuged for 10 min at 3000 rpm/min and 4 °C; 10 mL of the supernatant was used to determine the EPS content via phenol–sulfuric acid method.

### Activity assays of *L. edodes* enzymes involved in polysaccharide synthesis

One milligram of fresh mycelia was washed three times with phosphate buffer (20 mM, pH 6.5), and ground into powder in the presence of liquid nitrogen. The powder was dissolved in 3 mL phosphate buffer (20 mM, pH 6.5), and centrifuged at 10,000 r/min at 15 min, 4 °C. The supernatant was applied to three-enzyme activity test. Assay mixtures were prepared according to the type of the enzyme (Table 1); the reaction systems contained 960 μL of assay mixtures and 40 μL of crude enzyme. After incubation at 30 °C for 3 min, OD values at 340 nm were measured. Enzyme activities were determined by measuring changes in absorbance at 340 nm with the extinction coefficient ɛ_420_ = 6220 M^−1^ cm^−1^, using NADH^+^ as the substrate. Activities are expressed in international units (U/mg). One M NAD(P)H oxidized by enzymes within 1 min is defined as an enzyme unit (Peng et al. 2016).

**Table 1:** Reaction mixtures of enzyme activity tests

Name of the enzyme	Reaction system	References
UDP-Glcpyrophosphorylase (UGP)	50 mM Tris–HCl buffer (pH7.8), 0.4 mM UDP-Glc, 14 mM MgCl_2_,4 U Glc-6-phosphate dehydrogenase, 0.4 mM NADP^+^, 2.1 U α-phospho-glucomutase, 4 mM inorganic pyrophosphate	Bernstein and Robbins (1965)
Phospho-Glcisomerase (PGI)	50 mM potassium phosphate buffer (pH 6.8), 5 mM MgCl_2_, 4 U Glc-6-phosphate dehydrogenase, 0.4 mM NADP^+^, 10 mM fructose-6-phosphate	Grobben et al. (1996)
α-Phospho-glucomutase (PGM)	50 mM tri-ethanolamine buffer (pH 7.2), 5 mM MgCl_2_, 50 μM Glc-1,6-diphosphate, 0.4 mM NADP^+^, 4 U Glc-6-phosphate dehydrogenase, 1.4 mM Glc-1-phosphate	Qian et al. (1994)

### Quantitative real-time PCR (qRT-PCR)

The total RNA of *L. edodes* mycelium was isolated using Trizol reagent (Sangon Biotech, Shanghai, China) following the manufacturer’s instructions. The purity and quantity of RNA samples were measured using a Nano spectrophotometer (ND-1000 Thermo Scientific, Waltham, MA, USA), and the integrity was checked by agarose gel electrophoresis. An amount of 1.5 μg total RNA was synthesized to cDNA using a reverse transcription kit (Tiangen, Beijing, China), following the manufacturer’s instructions. The synthesized cDNA was diluted 10 times with nuclease-free water and stored at − 20 °C.

Gene-specific primers for qRT-PCR were designed using Primer-Blast (https://www.ncbi.nlm.nih.gov/tools/primer-blast/) with the following criteria: amplicon size of 140–180 bp, GC percentage content approximately 55%, Tm at approximately 60 °C. The primer sequences are listed in Table 2.

**Table 2:** Sequences of the primers used for the qRT-PCR analysis

Gene	Accession number^a^	Primer name	Primer sequence (5′-3′)
*ugp*	MT106079	*LeUGP_964F*	GACGGCCAAGGGGTTATTCA
		*LeUGP_1128R*	TTGACCGTGGCTCAAAGAGT
*pgm*	MT106081	*LePGM_578F*	CCCATGCCGACGAATACAGA
		*LePGM_1128R*	GGTGTGAGCGTAGGTCAAGT
*pgi*	MT106080	*LePGI_1190F*	TCCATCAGGGCACCAAACTC
		*LePGI_1330R*	CGGTCTTACCGAAGGCCAAT
*Rpl4*	MT106078	*Rpl4_F*	AATCGTAGACACCGTCAGCG
		*Rpl4_R*	TGACGAAACGGCCAAGATGA

The qRT-PCR reactions were performed in 96-well plates with SYBR Green detection using an iCycler iQ5 thermo cycler by using the kits of Bio-Rad Company (California, USA). Each biological sample was amplified in three technical replicates. Each reaction included: 1 μL of tenfold diluted cDNA, 0.5 μL of each primer (1 μM), 10 μL Cham Q Universal SYBR qPCR Master Mix (Vazyme, Nanjing, China), and nuclease-free water to a final volume of 20 μL. Negative controls that did not contain cDNA were also included for each primer pair. Reactions were performed under the following conditions: 95 °C for 5 min, followed by 40 cycles of 95 °C for 15 s, 60 °C for 15 s, and 72 °C for 30 s. Melting curves were observed to ensure that there was only one amplified product. The qRT-PCR products were also confirmed by agarose gel electrophoresis and sequencing. The expression levels of genes (*pgi*, *ugp* and *pgm*) coding for the three enzymes mentioned above (Table 1) were normalized by using *Rpl4* (internal control) (Xiang et al. 2018). The relative expression level of each gene was calculated using the formula Y = 10^△Ct/3^ × 100% (Chen et al. 2012), where △Ct is the difference in the cycle threshold value of the target gene (*pgi*, *ugp* and *pgm*) and the *Rpl4* control. Mean values were obtained from three biological replicates.

## Results

### Determination of the optimum metal ion concentration and induction time

**Fig. 1:**
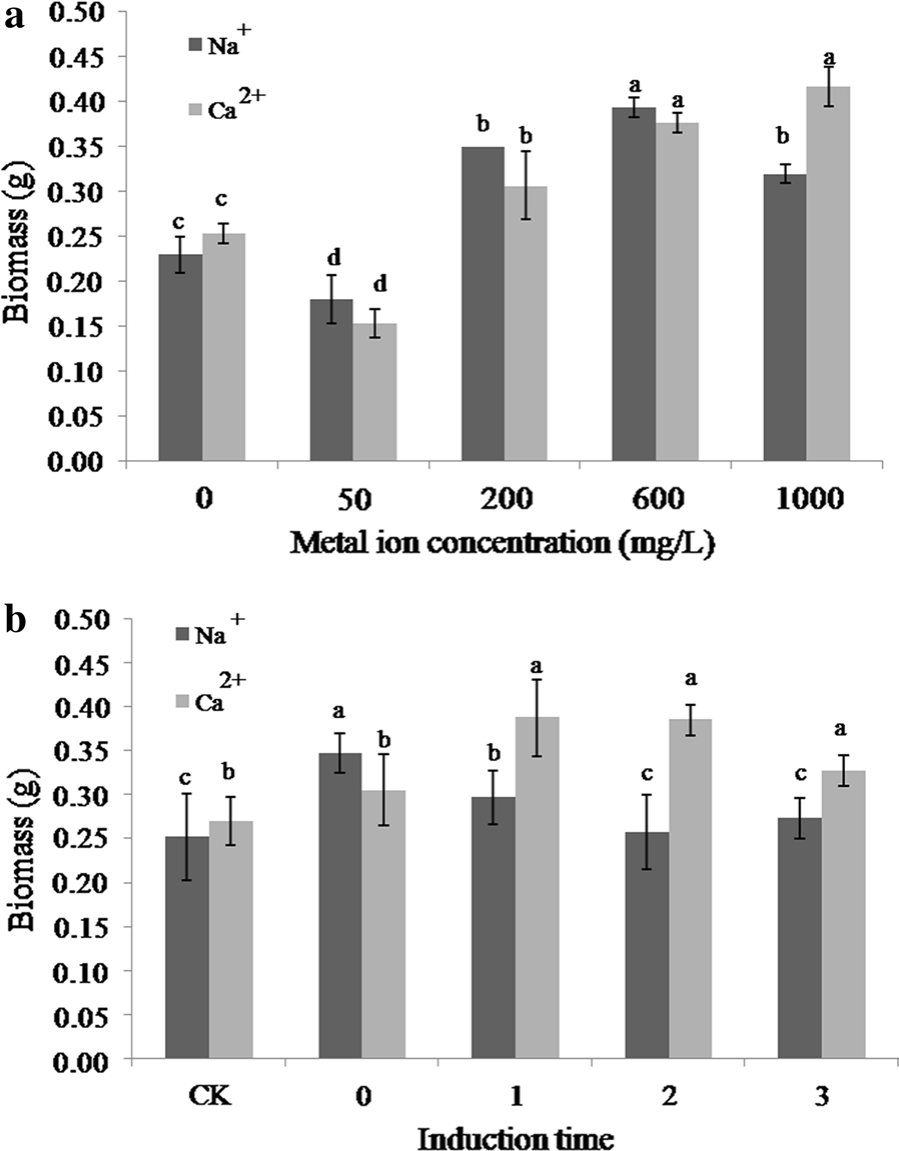
Mycelium biomass of *L. edodes* under different metal ions (**a**) and induction time (**b**). CK: control group, no metal ions added; 0: metal ions added during inoculation; 1: metal ions added after 3 days of static culture; 2: metal ions added after shaking for 7 days; 3: static after shaking culture metal ions added after 10 day. Different letters above bars indicate statistically significant differences between groups according to one-way ANOVA (n = 3, *p *< 0.05; refer in-text for test specifics and test statistics)

**Fig. 2:**
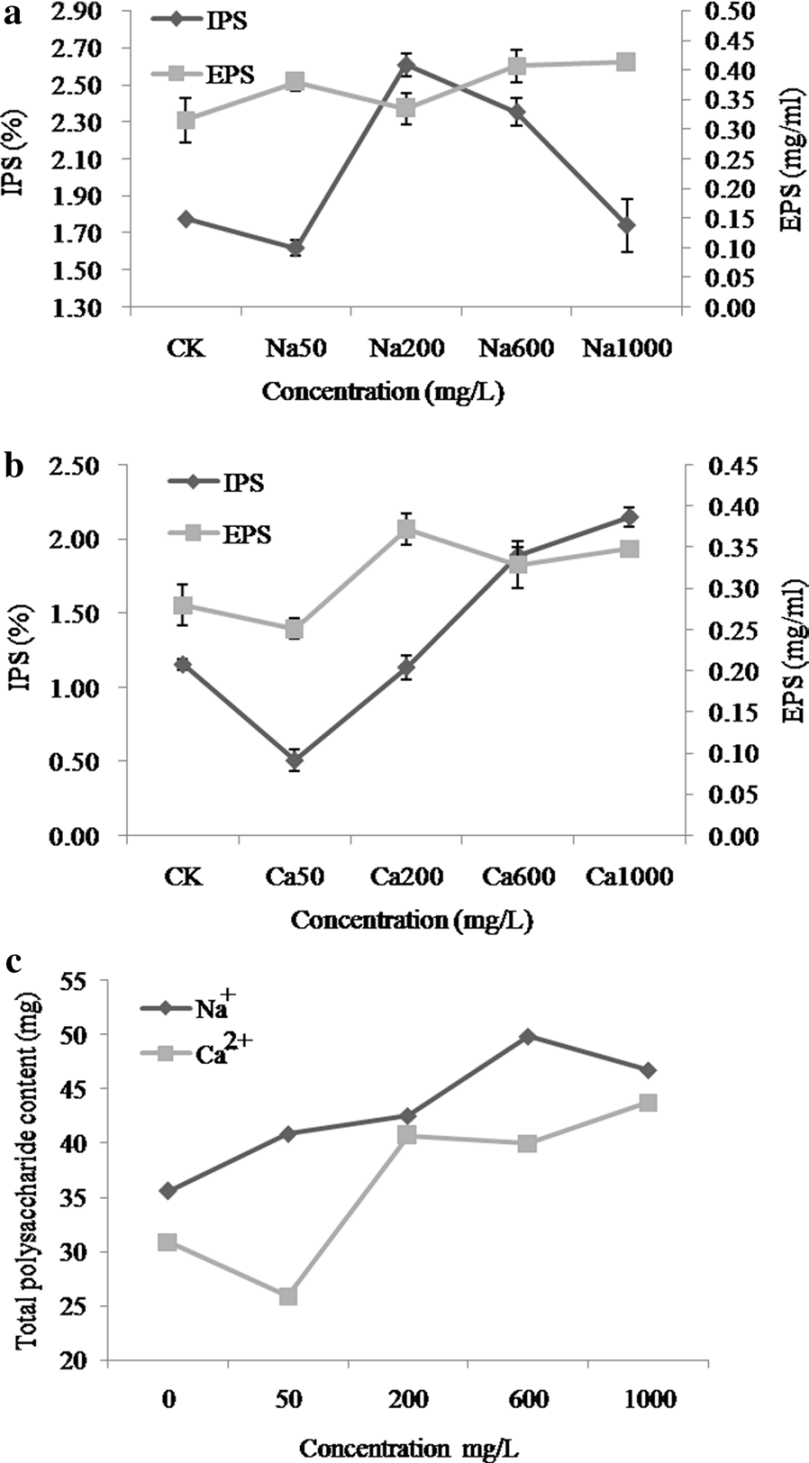
IPS, EPS under different concentrations of Na^+^ (**a**) and Ca^2+^ (**b**) and total polysaccharide (**c**)

Significant differences in mycelial biomass of *L. edodes* were observed in samples incubated with metal ion to that of control samples (Fig. 2). In the presence of low concentrations (50 mg/L) of Na^+^ and Ca^2+^, biomasses were 21.73% and 39.47% that of control samples, respectively. Higher concentrations effected biomasses in different ways. For Na^+^ treated samples, 600 mg/L was associated with 2.19-fold greater biomass yield than that of the control (Fig. 1a). For Ca^2+^ treated samples, mycelial biomass increased as the concentration of Ca^2+^ increased from 0.15 (50 mg/L) to 0.43 g (1000 mg/L). Samples incubated with metal ions had significantly different biomass yields during different periods of culture. The addition of metal ions at four time points were all associated with an increase in biomass. The greatest biomass yields were observed when metals were added at time point 0 (0.34 g at the inoculation time) and 1 (0.42 g after 3 days of static culture) for Na^+^ and Ca^2+^, respectively (Fig. 1b).

As the concentration of Na^+^ increased, different trends were observed in IPS and EPS contents (Fig. 2a). As Na^+^ concentration increased from 50 to 200 mg/L, the IPS content gradually increased; however, beyond 200 mg/L this content decreased. EPS content behaved antagonistically and increased with increasing concentration of Na^+^ (200–1000 mg/L). The greatest content of IPS (2.60%) and EPS (0.41 mg/mL) was observed at 200 mg/L and 1000 mg/L of Na^+^, respectively. Unlike Na^+^ treatment, changes in IPS and EPS content were similar under Ca^2+^ treatment (Fig. 2b). Compared to the control, low Ca^2+^ concentrations (50 mg/L) were associated with lower IPS and EPS; the highest IPS (2.14%) and EPS (0.37 mg/mL) concentrations were observed at 1000 mg/L and 200 mg/L Ca^2+^, respectively.

To further analyze the effect of Na^+^ and Ca^2+^ on polysaccharides, total polysaccharide content was measured (Fig. 2c). Under Ca^2+^ treatment, there was an increased trend of growth with the increase of concentration. Low Na^+^ concentrations were associated with lower polysaccharide yields; a trend of continuous increased growth was observed as Na^+^ concentration increased. The highest TPS content of 49.87 mg and 43.70 mg for Ca^2+^ and Na^+^, respectively, were observed at 600 and 1000 mg/L, respectively.

### Determination of optimal induction time

**Fig. 3:**
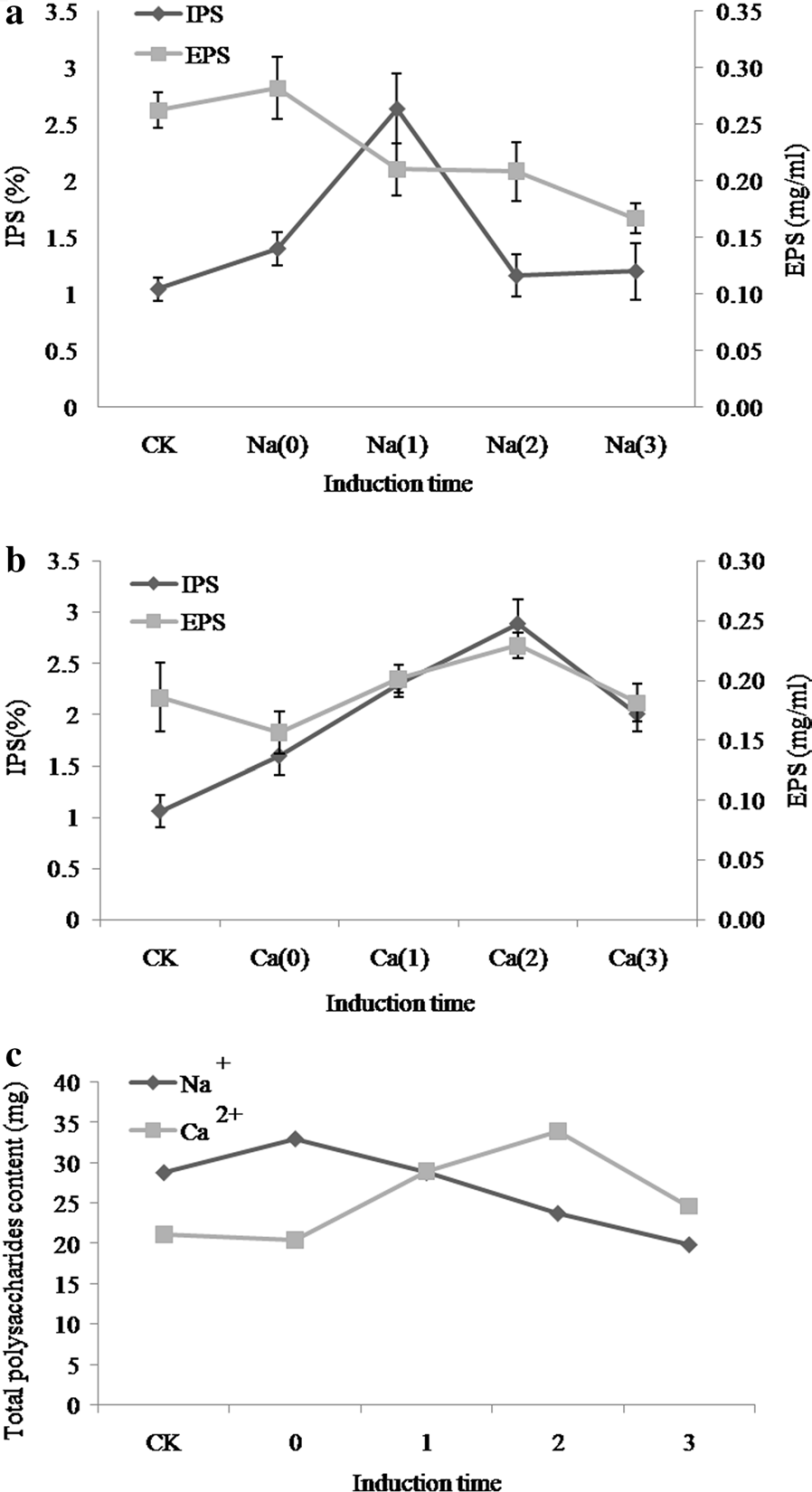
IPS and EPS content under different time periods Na^+^ (**a**), Ca^2+^ (**b**) and total polysaccharide (**c**). CK: control group no metal ions added; 0: metal ions added during inoculation; 1: metal ions added after 3 days of static culture; 2: metal ions added after shaking for 7 days; 3: static after shaking culture metal ions added after 10 days

Samples at different growth stages responded differently to external conditions. The additions of metals at different times were associated with significant differences in polysaccharide content (Fig. 3). Lower polysaccharide content was observed when Na^+^ was added in culture medium at later time points as compared to early time period. IPS content was greatest (2.63%) at stage 1 (Na^+^ metal ion added after 3 days of static culture), which was 60.52% greater than that of the control (Fig. 3a). On the other hand, the highest EPS content (0.28 mg/mL) was observed at stage 0 (Na^+^ metal ion added during inoculation) which was 6.87% greater than that of the control. Different trends were observed for Ca^2+^: maximum IPS (2.88%) and EPS (0.22 mg/mL) contents were obtained at a later induction time (stage 2) (Fig. 3b). Maximum TPS contents were observed at stage 0 (Na^+^, 33.02 mg) and 3 (Ca^2+^, 33.99 mg), which was 12.62% and 37.62% greater than that of the control, respectively (Fig. 3c).

### Dynamic changes in mycelial biomass

**Fig. 4:**
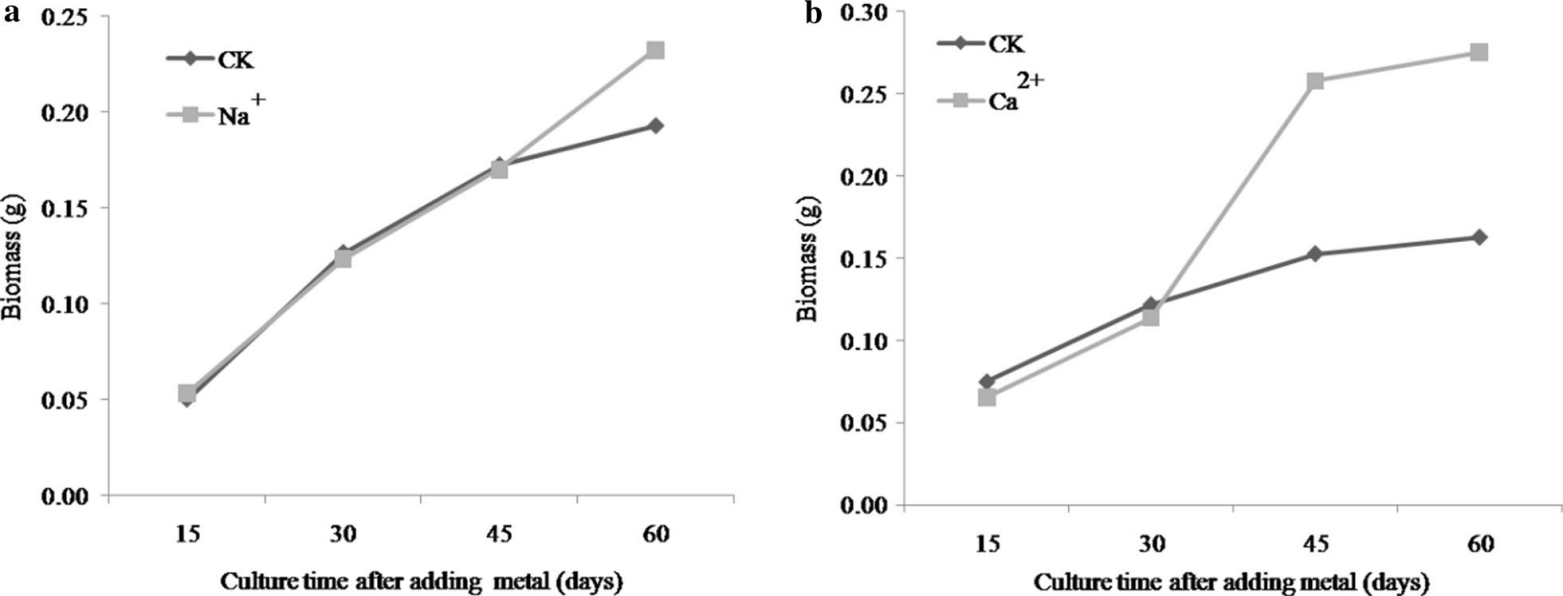
Dynamic changes of mycelial biomass under Na^+^ (**a**) and Ca^2+^ (**b**)

Na^+^ was associated with no significant dynamic changes in mycelial biomass from days 15–45, but a sharp dynamic trend was observed at days 45–60, which was 17.39% higher than that of the control group (Fig. 4a). Ca^2+^ was associated with a greater trend at 30 days and a continued enhanced growth was observed over the remaining culture period (Fig. 4b). The highest biomass value (0.27 g) was observed at day 60, which was 0.11 g greater than that of the control.

### Dynamic changes of mycelial IPS, EPS and TPS

**Fig. 5:**
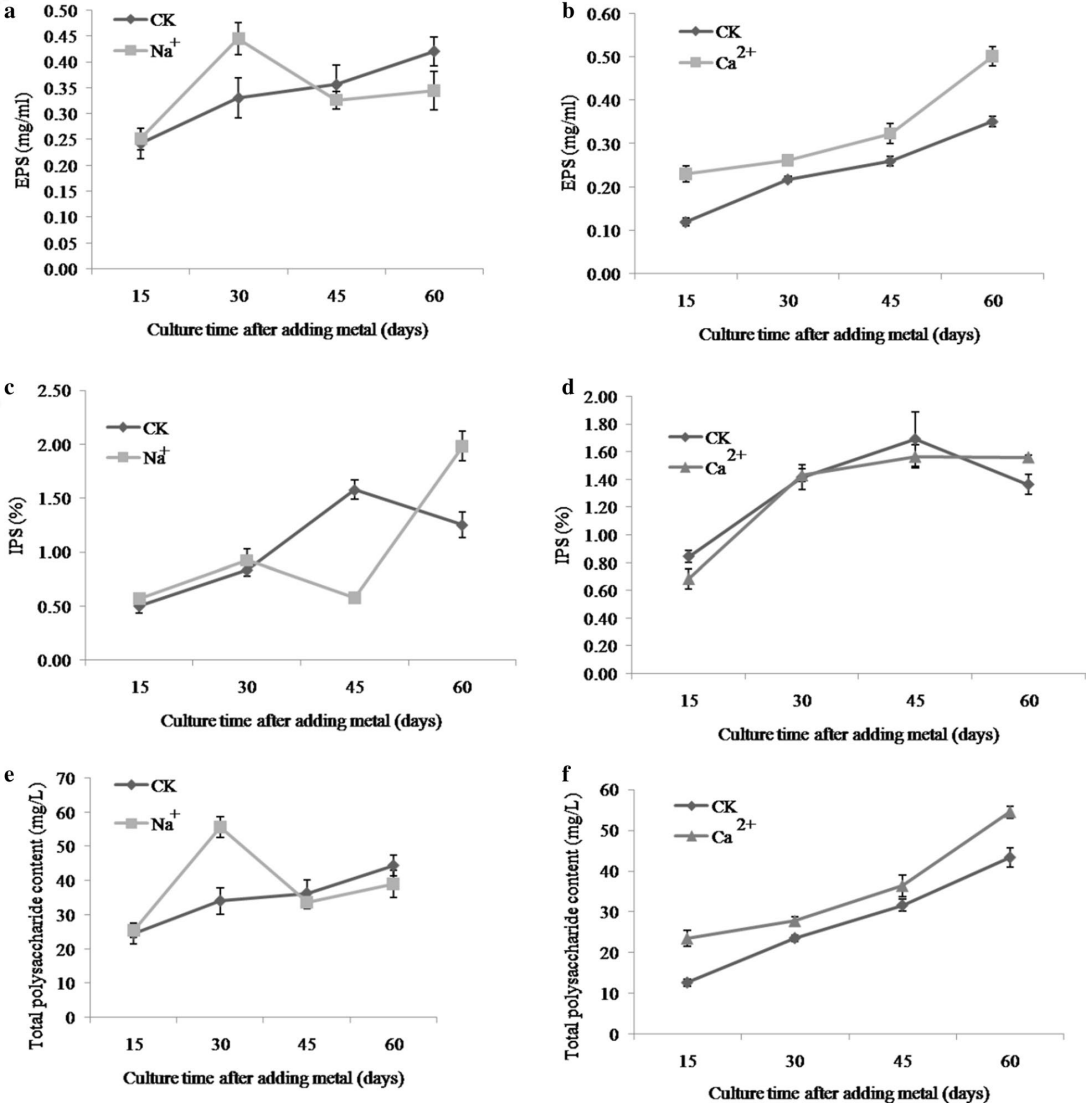
Dynamic changes in EPS (**a**, **b**), IPS (**c**, **d**) and TPS (**e**, **f**) at different time intervals under Na^+^ and Ca^2+^ metal ion treatments respectively

**Fig. 6:**
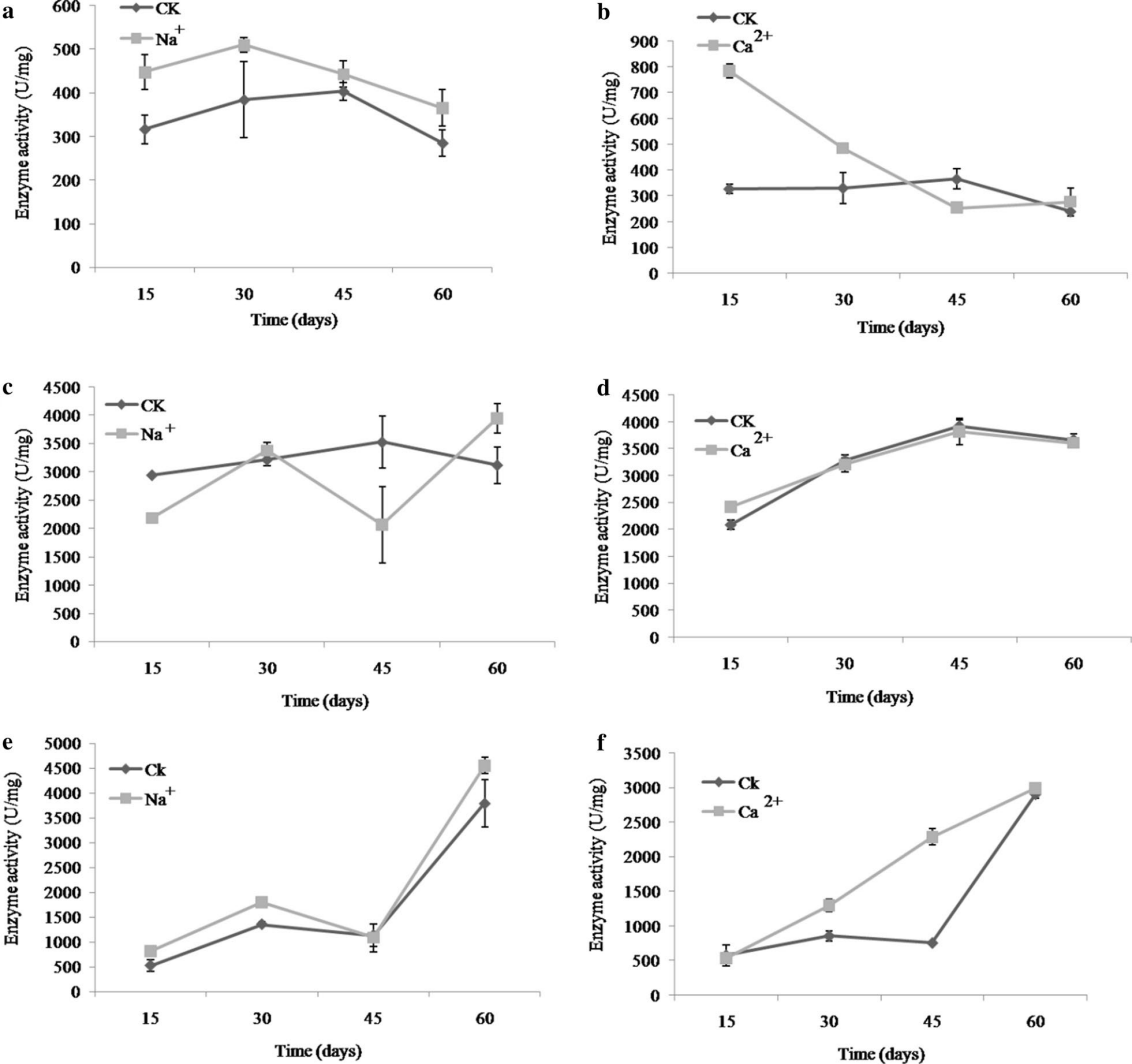
Enzyme activities of PGI (**a**, **b**), PGM (**c**, **d**) and UGP (**e**, **f**) under different culture periods and metal ions Na^+^ and Ca^2+^, respectively. 15 days, 30 days, 45 days and 60 days represent the incubation time after the addition of metal ions

Polysaccharide content responds in different ways to the presence of Na^+^ and Ca^2+^ (Fig. 6). In case of Na^+^ addition, EPS content at day 30 was 44 mg/mL (30 days), which was 25% greater than that of the control. However, as the culture time increased, the EPS content increased continuously in control samples, but decreased under Na^+^ treatment (Fig. 5a). Fluctuating EPS contents were observed under Ca^2+^ treatment, but overall Ca^2+^ treatment was associated with an increasing EPS content. Under Ca^2+^ treatment, EPS content was higher than that of the control for the entire culture time; EPS was highest at 0.50 mg/mL Ca^2+^ (60 days), which was 30% greater than that of the control (Fig. 5b).

In contrast to effect of Na^+^ on the IPS, there was a little increase from days 15–30 but it showed decreased trend at (30–45 days) of culture (Fig. 5c). However, at later stages from days 45–60 it produced highest IPS content (1.98%) at day 60, which was 34.21% more than that of control samples. Interestingly, the addition of Ca^2+^ had little effect on IPS content at day 60 (Fig. 5d). The highest TPS content (55.63 mg/L) was observed at day 30 of culture under Na^+^ treatment, which was approximately 38.7% higher than that of the control (Fig. 5e). Under Ca^2+^ treatment, TPS content was higher than that of the control at all points tested, and that promotion effect was not much different with continued culture time (Fig. 5f).

### Effects of metal ions on the activity of three key enzymes

Under Na^+^ treatment, changes in PGI activity were the same as those of the control, but it is higher than that of the control. Maximal enzyme activities were 409.97 and 384.04 U/mg for Na^+^ treated and control samples, respectively, tested at 30 days (Fig. 6a). In contrast to Ca^2+^ treated samples, the enzyme activity of PGI did not change much in control samples, while a rapid decrease trend was observed for days 15–45 (Fig. 6b). The highest PGI activity was observed at the beginning of Ca^2+^ treatment.

In the early stage of Na^+^ treated culture, PGM activity was lower than that of the control, and reached a minimum value (2057.84 U/mg) at day 45, which was 71.22% less than that of the control. However, PGM activity increased rapidly later, for day 45–60 and attains maximal enzyme activity 3937.83 U/mg at day 60 (Fig. 6c). Under Ca^2+^ treatment, the overall trend for PGM activity was similar to that of the control; no significant difference was found. In the early stage of culture at 15th, PGM enzyme activity continued to increase as culture time increased, and latter was slightly reduced (Fig. 6d).

Under Na^+^ treatment, the overall trend in UGP activity was similar to that of the control; no significant difference was observed between these conditions. The UGP enzyme activity increased rapidly after the 45th day of culture, and reached maximal activity (4553.35 U/mg) at the 60th day, which was 16.67% greater than that of the control (Fig. 6e). In Ca^2+^ treated samples, UGP activity was greater than that of control samples; maximal activity (2989.40 U/mg) was observed at day 60 of incubation, which was 2.79% greater than that of control samples (Fig. 6f).

### Effect of metal ions on the transcriptional expression of three key enzyme genes

**Fig. 7:**
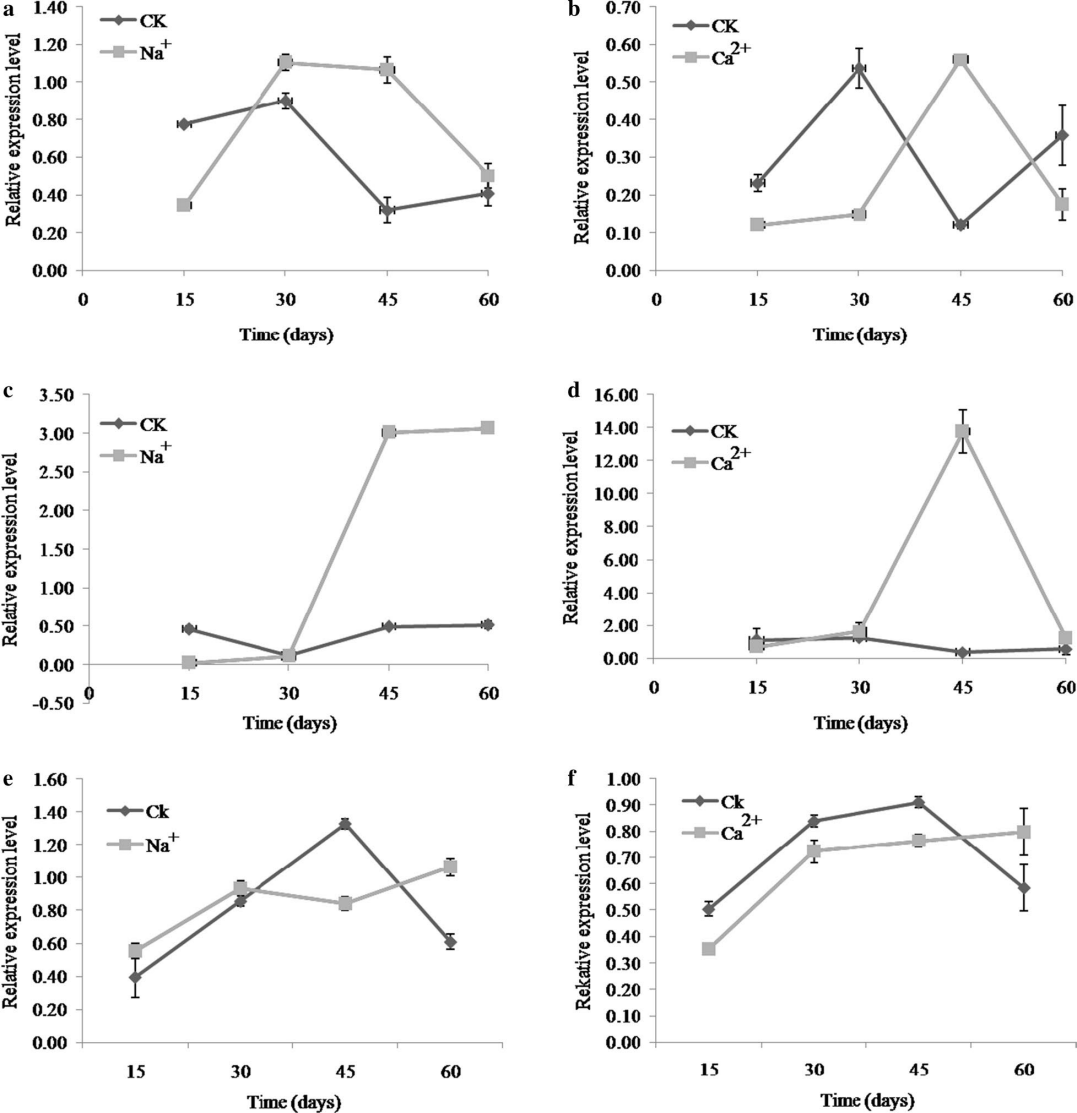
Transcriptional expression of *pgi* (**a**, **b**), *pgm* (**c**, **d**) and *ugp* (**e**, **f**) gene in different culture periods and metal ions Na^+^ and Ca^2+^. 15 days, 30 days, 45 days and 60 days represent the incubation time after the addition of metal ions

Under Na^+^ treatment, the relative expression level of *pgi* was lower than that of control at the day 15, but it rapidly increased later from day 20–30, and reached the highest expression level when cultured for 30 days (Fig. 7a). The biggest difference in enzyme activity was observed at day 45, which is 3.3 times greater in Na^+^ treated samples than that of control samples. Under Ca^2+^ treatment, the lowest expression levels of *pgi* were observed at day 15, which was 3.6 times less than that of control samples. Maximal *pgi* expression was observed at day 45 in Ca^2+^ treated samples, which was 4.7 fold greater than that of control samples (Fig. 7b).

The relative expression level of *pgm* did not change significantly in control samples throughout all the time periods for 15–60 days, while a higher expression level was observed under Na^+^ treatment (Fig. 7c). There was a rapid change between days 30 to 45 and the relative expression level under Na^+^ treatment which was 6.1 fold greater than that of the control at day 45 days. Following the same trend as Na^+^ treatment, under Ca^2+^ treatment, the highest expression level of *pgm* was observed at day 45 days; expression was 7.8 times greater than that of the control, expression levels decreased rapidly at day 60 (Fig. 7d).

Before day 45, the expression level of *ugp* showed little change or was expressed less under Na^+^ treatment; however, at later days of culture (45–60), the expression level increased as compared to control (Fig. 7e). Similar to Na^+^ treatment, under Ca^2+^ treatment, the transcriptional expression of *ugp* was less than that of the control. These results reveal that *ugp* expression was suppressed in the presence of Na^+^ and Ca^2+^ (Fig. 7f).

### Correlation analysis between PGI, PGM and UGP enzyme activities and polysaccharide content

The correlation coefficient between the three enzymes and polysaccharide content indicates that (Table 3), under Na^+^ and Ca^2+^ treatment, the IPS content was closely related to the activities of PGM and UPG. Moreover, under Na^+^ treatment, the IPS content correlated negatively with PGI activity. No notable correlations were observed between EPS content and PGI and UGP activities under Na^+^ treatment, but EPS and TPS content significantly correlated with the activities of UGP under Ca^2+^ treatment.

**Table 3:** Correlation coefficient between polysaccharides content and PGI, PGM and UGP activities

	IPS		EPS		TPS	
	Na^+^	Ca^2+^	Na^+^	Ca^2+^	Na^+^	Ca^2+^
PGI	− 0.980*	0.472	− 0.441	0.738	− 0.461	0.770
PGM	0.899	0.959*	0.591	0.639	0.632	0.693
UGP	0.998**	0.843	0.265	0.920	0.276	0.946

* Significant correlation at *p *< 0.05

** Significant correlation at *p *< 0.01

## Discussion

Lentinan is a bioactive compound that has been applied as a treatment for many human diseases (Pandya et al. 2019). Culture conditions such as metal content, can affect the growth, production, monosaccharide composition, and molecular weight of fungal polysaccharides, and thereby influence their biological activity. Various studies have been carried out to improve the polysaccharide yield and biological activity. In this study, mycelial biomass, and intracellular and extracellular polysaccharide content were significantly influenced by Na^+^ and Ca^2+^ in submerged cultures of *L. edodes*. In the case of dynamic changes, EPS content was 25% and 30% greater in the presence of Na^+^ and Ca^2+^, respectively, than control samples. IPS content was 36% and 12% greater for Na^+^ and Ca^2+^ treated samples, respectively, than that of control samples. Under the optimal NaCl concentrations (3 g/L), EPS content was 32.27% greater, and IPS content was 16.89% less than that of control samples in *P. linteus* (Zou et al. 2006). The mycelial growth of *C. militaris* was enhanced by K^+^, Ca^2+^, Mg^2+^, and Mn^2+^, but the EPS production only increased in media containing Mg^2+^ and Mn^2+^ (Cui and Zhang 2012). Further, the production of ganoderic acid was enhanced by the addition of Na^+^ and Ca^2+^ in *G. lucidum* (Xu and Zhong 2012; Xu et al. 2013). These results indicate that different metals have different effects on the growth and metabolism of different fungi. Fungi with different growth stages can respond differently to environmental changes, depending on growth stage. The optimal induction time of NaCl fermentation medium was observed at middle growth stage for *G. lucidum*, which significantly enhanced its ganoderic acid content (Xu et al. 2013). In the present study, for Na^+^, the highest total polysaccharide content was obtained when Na^+^ added at time stage 0 (metal addition at inoculation time), while for Ca^2+^; this was after shaking for 7 days.

At the early stages of fermentation, no significant dynamic changes were observed for mycelial biomass and polysaccharide contents. As growth time increased, the maximal biomass and polysaccharide contents were observed. The polysaccharide synthesis pathway is a complex metabolic process involving many enzymes (Jiang and Wu 2011). In this study, Ca^2+^ addition enhanced PGI and PGM enzyme activity, whereas Na^+^ increased UGP activity. The results from (Zhu et al. 2016) showed that higher activities of PGM, UGP, and PGI were desirable for the rate of IPS biosynthesis in *C. militaris.* The activities of a-PGM and UDPase were highly correlated with the amount of polysaccharide produced in *S. thermophilus* and *A. pullulans* (Degeest and De Vuyst 2000; Pan et al. 2013). Consistent with these reports, the IPS content was also significantly correlated to PGI, PGM and UGP enzymes under Na^+^ and Ca^2+^ treatments, while EPS was correlated with the activity of PGM in this study. This result was similar to the observation of a positive correlation between PGM activity and polysaccharide biosynthesis in *G. lucidum* (Tang and Zhong 2002).

The addition of Na^+^ and Ca^2+^ ion influenced the expression of several genes that involved in ganoderic acid biosynthesis (Xu and Zhong 2012; Xu et al. 2013). The transcription levels of *pgm*, *ugp*, and *pgi* involved in polysaccharide biosynthesis were analyzed in this study. Up-regulation of *pgm* and *pgi* were observed at day 45 under Na^+^ and Ca^2+^ treatment compared to the control, while *ugp* expression was up-regulated in later culture stages (60 days). This result is in agreement with a previous result that *ugp* was significantly up-regulated at late growth stage (Hertzberg et al. 2001). These results indicate that *pgm*, *ugp*, and *pgi* play key roles in the polysaccharide biosynthesis process in *L. edodes*. Among the three key genes, the expression levels of *pgm* were relatively higher than that of *pgi* and *ugp*, indicating that *pgm* may be a key gene for controlling the polysaccharide biosynthesis in *L. edodes*. This result is consistent with previous reports that *pgm* gene plays key roles in polysaccharide biosynthesis in *S. thermophilus* LY03 and *C. militaris* (Zhu et al. 2016).

## Data Availability

All data obtained have been included into the manuscript.

## Abbreviations

ACCC: Agriculture Culture Collection of China
BLAST: Basic local alignment search tool
Bp: Base pair
cDNA: Complementary DNA
EMP: Embden–Meyerhof–Parnas
EPS: Extracellular polysaccharide
GC: Guanine–cytosine
IPS: Intracellular polysaccharide
NCBI: National Center for Biotechnology Information
OD: Optical density
PDA: Potato dextrose agar
PGI: Phospho-glucose isomerase
PGM: Phosphoglucomutase
qRT-PCR: Quantitative real-time PCR
RNA: RiboNucleic Acid
rpm: Revolutions per minute
TPS: Total polysaccharide
UGP: UDP-glcpyrophosphorylase
